# The relationship between personal and professional goals and emotional state in academia: a study on unethical use of artificial intelligence

**DOI:** 10.3389/fpsyg.2024.1363174

**Published:** 2024-03-27

**Authors:** Ayhan Dolunay, Ahmet C. Temel

**Affiliations:** ^1^Faculty of Communication, Grand Library, Near East University, Nicosia, Cyprus; ^2^Grand Library, University of Kyrenia, Kyrenia, Cyprus

**Keywords:** academia, ethics, academics, emotions, artificial intelligence

## Abstract

Artificial Intelligence (AI) is a concept that has been a subfield of computer science since the 1950s. In recent years, with its growing development power, AI technologies have made significant progress and are now being used in many fields. Like in all areas, the use of AI technologies in academia has provided convenience to academics while also bringing ethical debates. In the literature part of the study, concepts such as AI, academia, academics and academic progress, ethics, ethical theories, academic ethics, and emotional states have been thoroughly examined and defined. In this study, starting from AI and scientific ethics, ethical issues arising from emotional states in academic research have been identified, and concrete solutions to these ethical issues have been proposed. The aim is to discuss the views of academics in order to determine what types of scientific ethical violations and prevention methods are involved. In this context, the semi-structured interview technique, which is one of the qualitative research methods, was preferred as the method. In the study, in-depth semi-structured interviews were conducted with 4 ethics experts and 4 psychology experts selected through snowball sampling technique. The data obtained through semi-structured in-depth interviews will be analyzed using content analysis. Within the context of the literature review and interviews: Ethics is based on the foundation of acting correctly. In this context, scientific ethics can be summarized as acting truthfully and honestly, not distorting data, and not trying to progress unfairly. The use of AI in academia is becoming increasingly widespread. From a positive perspective, this usage significantly contributes to making studies more practical. However, it can lead to problems such as unfair authorship, devaluation of human authorship, and incorrect data. The connection between academics’ professional advancement goals and emotional states becomes prominent in this context. The potential of AI to facilitate progression can lead to unethical use. To prevent such situations, it is recommended to organize training sessions to increase professional awareness, internalize ethics personally, establish ethical committees specific to the field of AI, conduct more effective audits by academic publication and promotion committees, and implement specific regulations for AI. Finally, for future academic studies, it is suggested that the usage of AI in academic research be measured and evaluated by ethics experts. For psychologists, conducting surveys with academics to explore how they use AI in the context of their emotional states and professional advancement goals is recommended.

## 1 Introduction

Digitalization, as in every field, has led to the progressive development and transformation of AI. Similar to many other fields, AI has undergone significant changes from its emergence in 1956 (Kokina and Davenport, 2017) to the present day (See Chiu et al., 2023). This autonomy and development, as all aspects of life, have various implications in academia. These effects have sparked numerous debates, both positive and negative perspectives.

Many tasks previously performed by humans can now be carried out by machines and algorithms. For example, tasks such as article segmentation, analysis, and data processing can now be done more quickly and effectively with the help of AI (Mijwil et al., 2023). As a result, there has been a transformation process in the academic world.

This transformation process directly affects the professional progression and emotional state of academics. On one hand, the use of AI allows for faster and more efficient work, but on the other hand, these technological advances have caused academics to question their roles and abilities and redefine themselves. The tasks performed by AI have prompted academics to question the topics they have previously worked on and have led to changes in research areas (Altıntop, 2023).

On the other hand, with the increasing use of AI, various debates have emerged in the academic world. While AI provides great convenience to academics in areas such as topic suggestions, editing sections, data analysis, it also raises concerns in areas such as knowledge sharing, the threat of eliminating human authorship, unethical behavior, misinformation, creativity, and human-specific skills. Concerns such as the replacement of humans and the decrease in the human factor have sparked debates among academics (Crompton and Burke, 2023).

This study specifically examines the connection between the use of AI and the professional progression and emotional state of academics. Taking into account the advantages and disadvantages brought about by the use of AI, the study aims to analyze the impact on academics’ career development and emotional state. This study is an important step toward better understanding the changes brought about by the use of AI in the academic world and discussing possible future impacts.

And specifically conducts research and discussion on the connection between the use of AI, the professional advancement of academics, and their emotional states.

## 2 Literature review

### 2.1 Basic concepts

#### 2.1.1 Artificial intelligence

AI refers to the ability of computers or computer-assisted machines to perform high-level logical processes that humans are capable of. These abilities include finding solutions, understanding, deriving meaning, generalizing, and learning (Öztürk and Şahin, 2018).

AI is a concept used to give computer systems human-like capabilities. AI enables computer systems to analyze tasks similar to human intelligence, including analysis, learning, problem solving, and decision making (Öztürk and Şahin, 2018).

The history of AI is quite extensive. The Dartmouth Conference in 1956 is considered the birthplace of AI. Since then, AI has rapidly developed worldwide. The concept of AI was popularized in a letter proposed by John McCarthy, Marvin L. Minsky, Nathaniel Rochester, and Claude E. Shannon during the Dartmouth Conference in 1956. Although John McCarthy is mentioned as the creator of this concept, AI was evaluated as an important step in the birth of AI in the offer letter (Arslan, 2020).

In the early stages, AI technology was limited to large computer systems and specialized software. However, nowadays, AI is being used in various fields such as mobile devices, smart home systems, automobiles, healthcare, and education (Öztürk and Şahin, 2018).

The applications of AI are vast. AI can be used in e-commerce websites to track customer behavior, in the financial sector for credit risk analysis, in traffic management, in the healthcare sector for diagnosis and treatment planning, and in automated factories, among many other fields. Furthermore, AI technology also makes it easier for people in their daily lives. You can use voice commands to quickly search on smartphones, create personalized music playlists, control devices at home using voice commands, and even have your emails written for you (Arslan, 2020).

While AI is used in many different fields, it also has significant effects in academia. At this point, it would be appropriate to briefly define the concepts of academia and academics.

#### 2.1.2 Academia, academics, and academic advancement

The origin of the word “academia” is attributed to Plato’s school named “Akademia” near Athens. Today, the word “academia” is used interchangeably with the word “university,” which is defined as an educational institution that is centered around science and where knowledge is produced and disseminated. However, there are differences in meaning and function between Plato’s Akademia and today’s universities. The emergence of the first universities took place after the 11th century, and over time, the functions and expectations of universities have diversified (Akcan et al., 2018).

Universities have undertaken various roles throughout history (Aydin, 2016: 14–20). The prominent ones among these roles are: generating knowledge, disseminating knowledge, providing professional education, imparting general culture, serving the community, and finally, being an actor in the global economy. Gasset (1998, 52) argues that universities have three main tasks: to ensure the cultivation of any individual, to provide the necessary knowledge and experience to perform any profession, and to train researchers (Akcan et al., 2018).

Academia forms the foundation of the concepts of academician/academics. In the process that has survived from the past to the present, this term began to be used for a certain career and the individuals who hold this career (Gürkan, 2018).

An academician is someone who has received undergraduate education in a discipline, gained expertise by pursuing a graduate education in the same or a different discipline, and works at a university. Academic career is a type of career that offers a wide range of opportunities, goes beyond a specific job, and represents not only work but also a way of life and thinking (Gürkan, 2018).

The academic advancements or promotions of academics are also important in terms of the subject matter. There are three evaluation methods commonly used and considered appropriate for academic promotions (Demir et al., 2017): *Academic publications and citations received, Practices in education and teaching, University and community service.*

For example, in promotions to the rank of associate professor in Turkey, oral examinations are also used as a criterion in addition to these. Generally, globally, measurement and practice are widespread, with the highest emphasis on the first category. Academic advancements require a meticulous examination and adherence to ethical rules (Demir et al., 2017).

The evaluation method commonly used in academic advancements is the academic publications of academics and the citations they receive. In this method, the number, quality, and impact of articles published by an academician, as well as the number of citations.

### 2.2 Ethical theories and their adaptation to academic ethics

Setting aside the definition and boundaries of the concept of ethics, briefly exploring the approaches of philosophers to ethics throughout the ages will contribute significantly to explaining the current understanding of ethics and academic ethics. This is because ethics, being a concept that has existed for centuries, still holds great importance.

Socrates, who lived in Athens between 461 and 399 BCE, endeavored to educate the people of Athens on ethical matters. He emphasized the significance of knowledge in making ethical decisions, pointing out that ignorance is one of the main causes of wrong decisions. Applied to academics, this implies that scholars can make ethical decisions only when they are knowledgeable about the subject at hand, maintaining its validity in contemporary times.

According to Plato, a student of Socrates, virtues such as moderation, courage, and wisdom come together to create the highest virtue, forming justice. Plato’s concept of justice is broader than the contemporary understanding, signifying a moral-good life seen as the ultimate good (Peck and Reel, 2013: 9; Dolunay, 2018). In other words, to achieve a good life, one must obtain a morally good life. This attainment, for academics, can contribute to the awareness of society and individuals.

Aristotle, significantly influenced by Socrates and Plato, believed that ethical decision-making is a skill (techne) and that ethical behavior cannot be a precise science because there is no formula that fits every situation. Aristotle also advocated avoiding extremes. He viewed virtue as a middle ground between excess and deficiency. Aristotle saw the acquisition of the right character through education as essential for making the right choices. Learning from books, intellectual virtues gained through reading ethical rules, is another aspect of Aristotle’s philosophy (Peck and Reel, 2013: 10; Dolunay, 2018).

Aristotle argued that ethical virtues are learned through actions and must be acquired as habits. This doctrine requires possessing the right character for ethical behavior. In the context of today, academics using data obtained through ‘unethical’ means in their academic research, exceeding ethical boundaries in the application of AI, can be considered an excess. In such situations, finding a middle ground between excess and deficiency by behaving virtuously becomes crucial. The 18th-century philosophers, Bentham defined the principle (basic utilitarianism and the utility theory) currently considered a classic approach in terms of pleasure and pain instead of benefits and harms (Peck and Reel, 2013: 13; Dolunay, 2018). When applied to academics, this theory highlights the necessity for scholars to balance individual progress in their academic field with the respect and reputation of academia. Individual progress is important for the development of the field, but academics must achieve this within legal and ethical boundaries.

In the 20th century, Ross believed in *prima facie* duties, including keeping promises (fidelity), showing gratitude for good, being fair, improving the lives of others (beneficence), avoiding harm, making amends when necessary (reparation), and self-improvement. Ross did not consider these duties as the only ones, allowing for the list to be expanded. In some ethical dilemmas, multiple duties may apply. In such cases, individuals must decide which duty takes precedence for that particular situation (Peck and Reel, 2013: 16; Dolunay, 2018).

Contemporary philosopher Rawls, a Harvard professor, created a concept of justice that many students find useful in ethical decision-making. In this context, a person should ignore their own position, placing themselves behind a veil of ignorance, and make decisions (Peck and Reel, 2013, p 17; Dolunay, 2018). Applied to academics, this theory suggests that an academic, when conducting research or publishing, should consider the potential harm to individuals or groups and the impact on academia and the requesting institution without being aware of their hierarchical position.

In conclusion, exploring the ethical perspectives of philosophers across different eras provides valuable insights into the current understanding of ethics and its application in academia.

### 2.3 Academic ethics and emotions of academics relations

The word “ethics” originates from the French word “éthique,” which in turn comes from the Old Greek word “ethios,” meaning character and moral. This term carries the meaning related to morality. The word “ethios” is derived from the Old Greek word “ethos,” which encompasses custom, morality, tradition, and manners (Dolunay, 2018: 26).

According to Pieper, “ethics is not only a theoretical scientific concept but also something that can be practically realized” (Uzun, 2007; Dolunay and Kasap, 2018). In other words, ethics does not have meaning on its own but gains significance when associated with something, such as academic ethics.

In scientific research, ethics refers to the moral principles and norms that scientists must adhere to in the research and publication processes. Scientific ethics aims to ensure the accuracy, reliability, and societal benefit of science. Adhering to ethical rules in scientific research enhances the reputation of both scientists and the scientific field (Yördem and Şeker, 2018).

Ethical rules in scientific research include: Truthfulness, diligence, transparency, impartiality, social benefit, education, appreciation, and avoiding ‘ethical violations, improper citations, fabrication, falsification, duplication, fragmentation, unjust authorship’ (Resnik, 2012).

On the other hand, all these ethical values, especially in the context of the academic advancement goals of academics, need to be carefully considered. While the significance of emotions and advancement goals has been discussed in various studies, it is crucial to avoid unethical approaches driven by emotions and advancement goals to achieve success more rapidly. Otherwise, with the influence of advancement goals and aspirations (negative emotional states), unethical situations such as unjust progress, persistent seeking of recognition and the spotlight in academia, desire to see one’s name frequently in academic publications (the Hollywood effect) can emerge (Ercan et al., 2021).

In order to be successful in the academic field, it is generally a vital goal for academics. The motivation and ambition necessary to achieve success can encourage academics to become better researchers, writers, or academics. However, it is a fact that an academic who cannot control their emotions may resort to unethical behavior for achieving success (Maya, 2013).

Ethics in the academic field is based on principles of honesty, impartiality, and respect. When conducting research, evaluating students, or preparing publications, academics should be objective and focus on universal knowledge and principles of justice instead of personal interests. However, an excessive desire for success can lead an academic to deviate from ethical principles (Tunç, 2007).

An academic lacking emotional control may try any means to compete with colleagues. They may use another academic’s work without permission, manipulate results, or work unfairly to gain an advantage over other researchers. Such behaviors can undermine trust in the academic field, affect the work of other researchers, and harm the scientific community (Maya, 2013).

These unethical behaviors prevent everyone in the academic field from feeling safe and in a fair environment. Respecting the value of everyone’s academic work is important to allow individuals to freely express their ideas and progress objectively (Maya, 2013).

In conclusion, it is normal to strive for success in the academic field, but an academic who cannot control their emotions may resort to unethical behavior. Academics need to be conscious of emotional control for the development of the academic community and equal opportunities for individuals. Upholding principles of objectivity, honesty, respect, and justice is important to avoid unethical behavior (Tunç, 2007).

At this point, especially with the involvement of the use of AI, the situation becomes more complex.

One of the most important developments in human history can be considered the rise of AI. While this technology has had a significant impact in various sectors, it has also caused important transformations in the academic field. However, although AI is a tool that supports and enhances people’s work, it can sometimes be subjected to unethical uses (Ülman, 2006).

An academic becoming excessively ambitious and losing control of their emotions in order to achieve success can also lead to the unethical use of AI. This situation presents behavior that contradicts ethical rules and human values in the scientific world. Considering that academics have a mission to produce knowledge, explore, and enhance the well-being of society, using AI unethically would be an approach that undermines this mission (Ülman, 2006).

An academic using AI negatively in order to achieve their goals is also contrary to the concept of scientific ethics. Science is a discipline that promotes objectivity, impartiality, and freedom of thought. Therefore, using a tool like AI, which supports scientific research, in the shadow of personal ambitions and emotions, both damages trust in science and misdirects knowledge construction (Altıntop, 2023).

Another unethical aspect of using AI in the shadow of an academic’s emotional state is the misuse of information. AI technologies, with their ability to perform big data analysis and make predictions, enable academics to quickly attain important results. However, an academic taking advantage of these rapid results by presenting fake data or manipulating results can cause great harm to the scientific community and society (Altıntop, 2023).

In this context, the pursuit of success in the academic field by academics losing control of their emotions and using AI unethically is an unacceptable situation from ethical, scientific, and societal perspectives. Academics must firmly adhere to ethical values while fulfilling their scientific responsibilities. Academics who combine the advantages provided by AI with ethical and human values will contribute to future scientific advancements and societal benefit (Altıntop, 2023).

In this framework, it would be appropriate to discuss academia and AI separately under a separate heading and briefly touch upon their positive and negative effects.

### 2.4 Artificial intelligence and academia

AI refers to the ability of computers or computer-supported machines to perform high-level logical processes that are typically associated with human capabilities. These skills include finding solutions, understanding, deriving meaning, generalizing, and learning (Muthukrishnan et al., 2020).

The term AI is used to describe the concept of giving computer systems human-like features. AI empowers computer systems to analyze, learn, solve problems, and make decisions in a manner similar to human intelligence (Muthukrishnan et al., 2020).

The history of AI is quite extensive. The Dartmouth Conference in 1956 is considered the birthplace of AI, where its foundations were laid. Since then, AI has rapidly developed worldwide. The concept of AI first emerged in a proposal letter presented at the Dartmouth Conference in 1956 by John McCarthy, Marvin L. Minsky, Nathaniel Rochester, and Claude E. Shannon. While John McCarthy is remembered as the creator of this concept, the proposal letter is considered a significant step in the birth of AI (Kokina and Davenport, 2017).

The use of AI is prevalent in various fields, including academia, serving various purposes. AI possesses capabilities such as teaching, structuring articles, conducting research and data analysis, and handling large-scale data examination and analysis, among other features (See Chiu et al., 2023).

#### 2.4.1 Positive perspective

AI has numerous positive aspects, and a few of them are outlined below:

Teaching at the university level: AI possesses the capability to instruct courses that require expertise in a specific subject. For example, Assistant Professor Dux at Near East University is an AI instructor. Through AI, students can more effectively access the courses they need (Mijwil et al., 2023).

Article structuring: AI can automatically divide a chosen topic into sections. By identifying key words or topics in texts, AI can also suggest titles and section headings. This allows for quick structuring of articles with less time investment (Mijwil et al., 2023).

Conducting analyses: AI has the ability to perform rapid and precise analyses on large datasets. For instance, an AI system can analyze uploaded text, images, or audio. These analyses assist in determining better strategies and making informed decisions (Mijwil et al., 2023).

Language translation: AI can translate text into multiple languages, facilitating communication among individuals who speak different languages. Additionally, it enables understanding of international articles or books in one’s own language (Lund et al., 2023).

#### 2.4.2 Negative perspective

One of the primary negative features of AI is the potential for unfair content generation. AI algorithms can generate new content by analyzing large amounts of data. However, there may be limitations on the accuracy and sensitivity of this content. AI can allow people to disseminate misinformation or produce inaccurate content (Sariyasa and Monika, 2023).

Another concern related to the use of AI is its potential for unethical behavior. People can use AI for wrongful purposes. For example, an individual or group that does not put in effort may use AI to generate content effortlessly and achieve success as a result (Thunström, 2022).

The advancement of AI poses a risk of eliminating human writing. AI algorithms can produce complex writings and reports, taking over tasks that many people currently perform. This situation could lead to unemployment in this field (Jabotinsky and Sarel, 2022).

Furthermore, AI can pose a threat to unfair promotion and academic progress. For instance, an automatically generated article or thesis that appears high-quality can be produced using AI. In such cases, many individuals may present these articles and theses as original works, leading to undeserved academic success. In summary, some of the negative features of AI include unfair content generation, unethical behavior, the risk of eliminating human writing, and the possibility of unfair progress and academic advancement. It is important to consider these concerns and regulate the development of AI (Thunström, 2022).

For example, there are significant debates in the literature about the negative and destructive effects of using AI in the field of education (Păvăloaia and Necula, 2023). These can be listed as technology addiction in education, the problem of determining responsibility in the event of a potential error, concerns about individuals losing their jobs, and issues related to data collection and analysis.

Technology Addiction: Individuals can become excessively dependent on AI-supported educational tools, deviate from traditional learning methods, and become detached from real-world interactions (Păvăloaia and Necula, 2023).

Responsibility Issue: When learning content or decisions provided by AI are incorrect, determining who is responsible can become uncertain. This situation may lead to disagreements on responsibility between educational institutions and technology providers (Sáiz-Manzanares et al., 2022).

Risk of Unemployment: If certain traditional teaching roles are taken over by AI and automation, teachers and other education professionals may face the risk of unemployment.

Inequality and Discrimination: AI algorithms can reflect biases and deepen inequalities in education. For example, equal opportunities may not be provided to students based on factors such as their ethnicity, gender, or socioeconomic status (Sáiz-Manzanares et al., 2022).

In order to address these issues, it is important to carefully establish governance, regulation, and ethical standards in the development and implementation of AI-supported education systems. Additionally, awareness of the risks associated with the use of AI technologies in education and continuous efforts to mitigate these risks are necessary.

## 3 Research

### 3.1 Method

In the study, the aim is to discuss the opinions of ethics experts and psychology specialists. In this context, the method chosen is the semi-structured interview technique, which is one of the qualitative research methods.

Interviews are used as a professional technique or auxiliary tool in many social science fields such as journalism, law, and medicine (Kahn, 1983 Tekin, 2006: 101). An extensively used data collection technique in qualitative research, interviews provide the interviewed individuals with the opportunity to express themselves directly, while also allowing the researcher to observe the interviewee comprehensively (McCraken, 1988: 9; Tekin, 2006: 102).

The interviewed individuals were asked questions covering all dimensions of the research topic, and detailed answers were obtained; it is a technique that enables the direct collection of information (Johnson, 2002: 106; Tekin, 2006: 102). Interviews can be categorized as unstructured, semi-structured, and structured (Punch, 2005: 166; Tekin, 2006: 104). Semi-structured interviews use predetermined questions, making them more limited compared to unstructured interviews, but it is possible to ask spontaneous questions and elaborate on targeted data/responses based on the course of the interview.

The data was collected through interview forms prepared by the authors of the study, containing 5 questions for psychology experts and 7 questions for ethics experts. Interviews were conducted between September 2023 and November 2023 over a period of 2 months. The interview forms were delivered to participants online, and they were asked to provide written answers to ensure no missing or lost responses. The collected data was archived in an online cloud database.

### 3.2 Sampling

The study involved semi-structured in-depth interviews with four ethics experts and four psychology specialists selected through the snowball sampling technique.

The presence of ethics experts in the interview group is primarily due to the need to discuss the ethical implications of AI usage. The development of AI technology has brought forth numerous ethical issues, necessitating the need to address or manage these issues. Therefore, by including ethics experts in the interview group, the aim is to discuss the emerging problems by obtaining views and recommendations on the use of AI and ethics in academia.

On the other hand, the inclusion of psychology experts in the sample is primarily aimed at investigating and understanding the potential emotional effects that may arise during the use of AI by academics. Unethical uses of AI are a significant factor in the emotional effects it may have on individuals in their daily lives and/or professional careers. Understanding and addressing such emotional states requires the insights and recommendations of psychology experts.

Thus, while ethics experts provide opinions and recommendations on the ethical use of AI, psychology experts will enrich the study with their responses on the emotional effects and mood states related to AI. Their collaboration enables a more comprehensive assessment of the ethical and emotional dimensions of AI, contributing to evaluating the potential consequences of unethical uses and fostering interdisciplinary work.

The snowball sampling technique was employed, selecting individuals based on their expertise in their respective fields and a minimum of 5 years of professional experience. Additionally, another important criterion was the selection of academics who have studies or knowledge in this area. Therefore, professionals with both professional experience in the field and knowledge through studies or research in the relevant field were selected for the study.

The selected individuals were asked to recommend others who meet these criteria. Among the experts in the field, the criterion of a minimum of 5 years of professional experience shaped the interview group, ranging from 5 to a maximum of 25 years of professional experience. The snowball sampling technique is a method that involves selecting a reference person related to the subject of the study and reaching other individuals through recommendations. This method is iterative, and participants guide researchers, contributing to the growth of the sample. Therefore, it is known as the “snowball effect” (Biernacki and Waldorf, 1981).

### 3.3 Analyses

The data obtained from semi-structured in-depth interviews were analyzed through content analysis. Content analysis is a research technique where valid interpretations extracted from the text are revealed through consecutive processes (Weber, 1990: 9; Koçak and Arun, 2013: 22). Depending on the context of a specific study, detailed coding may or may not be required (Yıldırım and Şimşek, 2008: 233; Karataş, 2017: 80).

In this context, due to the nature of the study, there was no need for intricate coding and theme formation. The themes and codes are as follows:

In the analyzes under the specified themes and codes (Table 1), 20% of the direct opinions of the interview group were included. The names of the participants are given in codes as P1, P2, P3, etc.

**Table 1:** Themes and codes.

Themes				
Codes	Terms of ‘ethics’ and academic ethics	AI in academy and positive–negative effects of using AI in academy	Relation between emotions of academics, AI and academics professional goals	ethical problems raised by AI and solution suggestions in the context of scientific ethics
	Terms of ‘Ethics’	AI in Academy	Emotional Situation	Ethical Problems
	Academic Ethics	Positive and Negative Effects	*Unfair Academic Professions and AI*	Solution Suggestions

## 4 Terms of ‘ethics’ and academic ethics

### 4.1 Terms of ‘ethics’

Ethics is a concept that is difficult to define. Generally, it can be defined as a research discipline where moral situations are described, observation tools are developed; criteria are constructed based on what is good and bad or what is right and wrong, and a critical demand where they are validated (Moressi, 2006: 23; Girgin, 2000: 144).

Similarly to this definition, the interview group has also provided a response in line with the prevailing consensus in the literature.

The interview group has collectively defined the concept of ethics as acting in accordance with the correct principles and behaving in accordance with professional fundamental principles:

P2: “*Determining what the correct way to act could be. In other words, how should one act?”*

P4: *“I define the concept of ethics as the identification of individual, professional, institutional, and societal values, and the use of these identified values as a criterion for evaluating human behavior.”*

### 4.2 Academic ethics

In scientific research, ethics refers to the moral principles and norms that scientists must adhere to in the research and publication processes. Scientific ethics aims to ensure the accuracy, reliability, and societal benefit of science. Adhering to ethical rules in scientific research enhances the reputation of both scientists and the scientific field (Yördem and Şeker, 2018).

The interview group also made a similar definition. Scientific ethics is defined in accordance with the concept of ethics as being focused on acting correctly, behaving honestly and fairly, working within boundaries that are beneficial and respectful to society and nature.

P1: *“All scientific research is conducted with the aim of finding truth, discovering new things, and finding solutions to observed problems. And while all of this is done, benefiting both the field and society, and humanity are fundamental goals; therefore, scientific ethics are indispensable.”*

P3: *“I evaluate scientific ethics in two ways. Firstly, individuals conducting scientific research should behave sensitively towards the environment and living beings related to the subject they are working on. In scientific research, to reach a conclusion, one should avoid behaviors that could harm the environment or cause physical or mental harm to living beings. Secondly, individuals conducting scientific research should not use any information or documents derived from previously conducted sources or sources of inspiration without citing references.”*

## 5 AI in academy and positive–negative effects of using Ai in academy

### 5.1 AI in academy

The use of AI is prevalent in various fields, including academia, serving various purposes (Chiu et al., 2023).

The interview group has expressed a common view that AI and AI technologies are used in the academic field. They have emphasized that the evolving and changing technology influences academia and that AI is utilized for both structuring and writing in academic research:

P2: *“I believe the application for AI in academic fields can be a way for a student, instructor, or researcher to begin their research….”*

P3: *“AI has recently contributed to both students’ and academics’ easy access to information, while also taking on an educational role with various developed AI modules.”*

### 5.2 Positive and negative effects

In academia, AI possesses capabilities such as teaching, structuring articles, conducting research and data analysis, and handling large-scale data examination and analysis, among other features (See Chiu et al., 2023). On the other hand, AI can allow people to disseminate misinformation or produce inaccurate content (Sariyasa and Monika, 2023). Same time, an individual or group that does not put in effort may use AI to generate content effortlessly and achieve success as a result (Thunström, 2022) AI algorithms can produce complex writings and reports, taking over tasks that many people currently perform (Jabotinsky and Sarel, 2022).

In accordance with the positive and negative aspects given above in the literature, the interview group has identified both positive and negative aspects of the use of AI in academia.

In this context, the positive aspects are as follows: time savings, easy access to resources, support in text writing, contribution to structuring and analysis, and assistance in creating visuals and tables:

P1: *“While AI provides us with many advantages like this, especially when conducting research, it saves time, facilitates cost, and perhaps brings us together with resources that may be difficult to reach. Therefore, I view its use in academic studies positively because it has many advantages in* var*ious positive aspects.”*

P4: *“Its positive aspects can assist scientists in finding academic sources in academic studies. It can help in applications that academics may not easily accomplish, such as data visualization.”*

On the other hand, negative aspects include: unfair authorship and/or devaluation of human authorship, the possibility of inaccurate data, plagiarism, and the potential for achieving results with minimal effort:

P1: *“As researchers, we should reevaluate the information provided by AI, strive to reproduce it, and look beyond the framework it presents to us. Of course, in addition to this, we should support the given information with our own ideas... Otherwise, the role of the researcher may deviate, various ethical issues may arise, the researcher’s image may be damaged, which may not be limited to the researcher alone but may also lead to questioning the discipline and credibility of the relevant field.”*

P4: *“As negative aspects, it can produce texts instead of academics. It can lead to plagiarism, and detecting it may not be easy.”*

## 6 Relation between emotions of academics, AI and academics professional goals

### 6.1 Emotional situation

Various studies in the literature indicate that there is a connection between mood states and the behaviors of academics from different perspectives (Maya, 2013). Similarly, the interview group holds the opinion that the mood states of academics are linked to their professional behaviors. However, within the focus of the study’s context, the interview group was asked for their opinions on whether academic advancement goals are also linked to mood states.

Questions about emotional states were directed specifically to psychology experts within the context of their expertise. The relevant group is of the opinion that the emotional states of academics are generally connected to their professional advancement goals:P5: “Ethics is a moral understanding in my opinion. Therefore, even if there are written rules, whether to comply with them or not is still within one’s personal discretion. Therefore, unfortunately, it is indeed possible to deviate from ethical rules within the framework of personal ambition and goals.”

*P5: “Ethics is a moral understanding in my opinion. Therefore, even if there are written rules, whether to comply with them or not is still within one’s personal discretion. Therefore, unfortunately, it is indeed possible to deviate from ethical rules within the framework of personal ambition and goals.”*

### 6.2 Unfair academic professions and AI

The interview group (psychology experts), expressing that emotional states and academic progress are interconnected, predominantly believes that, simultaneously, the influence of emotional states may lead to unfair use of AI in the context of academics’ career advancement goals. However, they also consider that some AI applications are not yet as competent in this regard:

*P8: “In the context of academics’ ambitions and advancement goals, unfair or unethical use of AI may be possible.”*

## 7 Ethical problems raised by AI and solution suggestions in the context of scientific ethics

### 7.1 Ethical problems

The ethical issues arising from the use of AI in academia include “the distortion and/or inaccuracy of data, unfair authorship, the formation of plagiarism, and reaching a correct or incorrect result without exerting effort.”

In this context, especially in the context of emotional states and career advancement goals, academics’ unjust use of AI driven by these motives can pose a significant ethical problem:

*P3: “The ethical issue arising from the use of AI in academic studies may occur when researchers present information derived from AI in their studies as if they had produced it themselves, rather than generating subjective knowledge.”*

*P4: “The most serious ethical issue is when an academic has their academic work done by AI. AI can easily generate data and interpret it into an article. Additionally, it can generate imaginary citations.”*

### 7.2 Solution suggestions

Those who provided recommendations against the ethical problems that may arise from unfair use of AI in the interview group have put forward the following solution proposals:

Implementation of professional awareness and training activities,

Individual internalization of ethical values and understanding that unfair progression is not appropriate in this context,

More careful evaluations by publication and/or academic promotion committees, utilizing more comprehensive technological control practices specific to the field,

Development/updates of ethical principles/rules in the context of AI,

Establishment of ethical committees specific to the use of AI.

*P1: “In this regard, scientific education programs should be organized, boards should conduct more active monitoring, and regulations need to be developed..”*

*P5: “...the peer (science) review board should be more meticulous in examining studies, and if they detect the use of AI, researchers should face more serious sanctions.”*

## 8 Findings and discussion

This study examines the relationship between the unethical use of AI in academia and the personal and professional goals, as well as the emotional states of academics. Findings obtained through interviews indicate various significant results.

Firstly, the findings of our research emphasize that scientific ethics is based on proper and honest conduct. In this context, scientific ethics involves acting honestly and accurately without distorting data and advancing with unjust motives. Ethics is based on the foundation of acting correctly. In this context, scientific ethics can be summarized as acting truthfully and honestly, not distorting data, and not trying to progress unfairly.

However, the increasingly widespread use of AI in academia poses new challenges to these ethical standards. As findings, it has been determined that artificial intelligence provides speed and practicality in academic studies. In particular, providing topic suggestions, determining the main sections of the studies, and contributing to analyzes provide significant convenience. On the other hand, it has been determined that the use of artificial intelligence in academic studies may also lead to negative situations such as reducing the value of human authorship, causing unfair authorship, and providing inaccurate data.

These findings are consistent with the views in the literature. While AI makes research more practical (Mijwil et al., 2023), it can also lead to issues such as unfair authorship, diminished value of human authorship, and incorrect data (Sariyasa and Monika, 2023).

On the other hand the research highlights the importance of the connection between academics’ professional advancement goals and emotional states While AI has the potential to facilitate progress, it can also lead to unethical use and weaken the integrity of academic research.

There are similar views in the literature on this subject. It is a fact that an academic who cannot control their emotions may resort to unethical behavior for achieving success (Maya, 2013).

However, due to the uniqueness of the subject of the study and the fact that it is a new field, opinions and suggestions regarding the relationship between the use of artificial intelligence and the emotional states of academics are not yet widely included in the literature. In this study, the claim that academics who cannot control their emotional state can achieve unfair success through the unfair use of artificial intelligence was also among the findings.

Within the framework of these findings, several concrete solution suggestions have been put forward. Firstly, continuous training sessions should be organized to enhance ethical awareness among academics and encourage personal ethical responsibility. Additionally, special ethical committees in the field of AI should be established, and academic publication and promotion committees should conduct more effective oversight. Furthermore, the development and implementation of specific regulations regarding the use of AI are crucial.

For future research, the involvement of ethical experts in evaluating the ethical consequences of AI use in academic research is essential. Moreover, psychology experts should conduct studies to better understand the relationship between academics’ emotional states and professional advancement goals with the use of AI.

Further studies are needed to better understand the ethical implications of AI use in academic research. Ethical experts evaluating the ethical aspects of AI use and contributing to the improvement of regulations in this area are crucial. Additionally, through survey studies conducted by psychology experts with academics, it may be possible to better understand the impact of AI on emotional states and professional advancement goals.

In conclusion, an approach addressing the ethical issues of AI use in academia should be adopted. This indicates the need for increased ethical awareness in the academic community, improved institutional regulations, and more research. Addressing the ethical challenges of AI use in academia requires a multidisciplinary approach integrating ethical principles, psychological perspectives, and institutional regulations. In this way, the benefits of AI use can be maximized, while potential risks can be minimized.

## 9 Instead of conclusion: “Academic ethics, emotions and future?”

Since the term was first used in academic articles, in the field of AI, significant changes and transformations have been observed, allowing the direct use of AI by individuals in various fields. For example, AI usage is becoming increasingly prevalent in health applications, personal mobile phones, computers, cars, and many other areas and products (Roser, 2023). Like all fields, the use of AI in academia has become evident in recent years, leading to serious debates. While studies highlighting the positive aspects of using AI in academia exist, there are also studies indicating its negative effects (Bakiner, 2023).

This study specifically addresses the use of AI in academia, with a focus on investigating the relationship between academics’ emotional states and unfair professional progression due to the use of AI.

The results of interviews conducted with ethics and psychology experts in this study lead to the following conclusions:

Ethics is based on the foundation of acting correctly. In this context, scientific ethics can be summarized as acting truthfully and honestly, not distorting data, and not trying to progress unfairly.

The use of AI in academia is becoming increasingly widespread. From a positive perspective, this usage significantly contributes to making studies more practical. However, it can lead to problems such as unfair authorship, devaluation of human authorship, and incorrect data.

The connection between academics’ professional advancement goals and emotional states becomes prominent in this context. The potential of AI to facilitate progression can lead to unethical use.

To prevent such situations, it is recommended to organize training sessions to increase professional awareness, internalize ethics personally, establish ethical committees specific to the field of AI, conduct more effective audits by academic publication and promotion committees, and implement specific regulations for AI.

Finally, for future academic studies, it is suggested that the usage of AI in academic research be measured and evaluated by ethics experts. For psychologists, conducting surveys with academics to explore how they use AI in the context of their emotional states and professional advancement goals is recommended.

## Data availability statement

The original contributions presented in the study are included in the article/supplementary material, further inquiries can be directed to the corresponding author/s.

## Ethics statement

The studies involving human participants were reviewed and approved by Near East University Scientific Research Ethics Committee. The participants provided written informed consent to participate in this study.

## Author contributions

AD: Formal analysis, Methodology, Project administration, Supervision, Writing – review & editing. ACT: Data curation, Resources, Writing – original draft, Writing – review & editing.
